# Field Plant Monitoring from Macro to Micro Scale: Feasibility and Validation of Combined Field Monitoring Approaches from Remote to in Vivo to Cope with Drought Stress in Tomato

**DOI:** 10.3390/plants12223851

**Published:** 2023-11-14

**Authors:** Filippo Vurro, Michele Croci, Giorgio Impollonia, Edoardo Marchetti, Adrian Gracia-Romero, Manuele Bettelli, José Luis Araus, Stefano Amaducci, Michela Janni

**Affiliations:** 1 Istituto dei Materiali per l’Elettronica e il Magnetismo (IMEM-CNR), Parco Area delle Scienze 37/A, 43124 Parma, Italy; filippo.vurro@imem.cnr.it (F.V.); manuele.bettelli@imem.cnr.it (M.B.); 2 Department of Sustainable Crop Production, Università Cattolica del Sacro Cuore, Via Emilia Parmense, 84, 29122 Piacenza, Italy; michele.croci@unicatt.it (M.C.); stefano.amaducci@unicatt.it (S.A.); 3 Integrative Crop Ecophysiology Group, Agrotecnio—Center for Research in Agrotechnology, Plant Physiology Section, Faculty of Biology, University of Barcelona, 08028 Barcelona, Spain; adrian.gracia@irta.cat (A.G.-R.); jaraus@ub.edu (J.L.A.); 4 Field Crops Program, Institute for Food and Agricultural Research and Technology (IRTA), 251981 Lleida, Spain

**Keywords:** phenotyping, tomato, RGB-based index, UAV, bioristor, multispectral, precision agriculture, sensors, vegetation indices, field monitoring

## Abstract

Monitoring plant growth and development during cultivation to optimize resource use efficiency is crucial to achieve an increased sustainability of agriculture systems and ensure food security. In this study, we compared field monitoring approaches from the macro to micro scale with the aim of developing novel in vivo tools for field phenotyping and advancing the efficiency of drought stress detection at the field level. To this end, we tested different methodologies in the monitoring of tomato growth under different water regimes: (i) micro-scale (inserted in the plant stem) real-time monitoring with an organic electrochemical transistor (OECT)-based sensor, namely a bioristor, that enables continuous monitoring of the plant; (ii) medium-scale (<1 m from the canopy) monitoring through red–green–blue (RGB) low-cost imaging; (iii) macro-scale multispectral and thermal monitoring using an unmanned aerial vehicle (UAV). High correlations between aerial and proximal remote sensing were found with chlorophyll-related indices, although at specific time points (NDVI and NDRE with GGA and SPAD). The ion concentration and allocation monitored by the index R of the bioristor during the drought defense response were highly correlated with the water use indices (Crop Water Stress Index (CSWI), relative water content (RWC), vapor pressure deficit (VPD)). A high negative correlation was observed with the CWSI and, in turn, with the RWC. Although proximal remote sensing measurements correlated well with water stress indices, vegetation indices provide information about the crop’s status at a specific moment. Meanwhile, the bioristor continuously monitors the ion movements and the correlated water use during plant growth and development, making this tool a promising device for field monitoring.

## 1. Introduction

Sustainable agriculture practices claim novel techniques to monitor plant growth, development and health with the aim of increasing crop yields to meet the demands of a rapidly growing population [1,2,3,4,5]. Several approaches can be applied to improve yields, reduce environmental threats and optimize input efficiency [6]. Recent approaches based on nanotechnology may improve in vivo nutrient delivery to ensure the precise distribution of nutrients as nanoengineered particles may improve crop growth and productivity, increasing fertilizer use efficiency [7]. The application of nanofertilizers (NFs) [8,9], nanoparticles [10,11] and organic compounds has shown promising results [12].

In this panorama, plant monitoring becomes central to sustainable agriculture. Technology and innovation can significantly improve the ability to monitor plant health during cultivation, thus fine-tuning farm management to improve agriculture sustainability [1,13].

Advances in precision agriculture (PA) technology can (i) significantly increase productivity, (ii) ensure high food quality and further decrease costs, and (iii) preserve crucial environmental resources [14,15]. Water savings, precision irrigation based on plant needs and the saving of natural resources are the main focus of PA [16] and are mandatory in view of the ongoing water crisis [17].

Several reviews describe platforms available for field monitoring and plant phenotyping on various observation scales [18,19,20,21,22,23,24,25,26]. Proximal and remote sensing (PRS) techniques are increasingly used for plant phenotyping because of their advantages in multi-dimensional data acquisition and analysis [27]. At the macro scale (aerial level), the rapid development of sensors and unmanned aerial vehicles (UAVs), imaging and data analysis algorithms, and improved computer capacities have enabled a broad range of possibilities for aerial precision farming to measure, for example, vegetation indices [28,29,30,31,32,33,34]. Overall, images have been demonstrated to be a good proxy for the characterization of quantitative plant traits [35,36,37,38]. At the medium scale, proximal RGB images can also be acquired through low-cost imaging methods to identify color indices to be used in crop management [39,40,41]. For crops such as wheat and maize, RGB images have shown similar or even better performance in comparison to a multispectral index like the Normalized Difference Vegetation Index (NDVI) in applications like predicting grain yield under different growing conditions, including water status [42,43,44] and availability of nitrogen [23,45,46,47] and phosphorous [41,48]. In the sensor panorama, a central role is played by soil monitoring sensors, with soil being crucial for plant development and yield and for improving the optimization of water resources [34,49]. 

Real-time sensing is now required not only to trace the time point index of plant health but also to trace plant health and growth dynamics [16,50,51,52,53,54]. To this end, a novel smart organic electrochemical transistor (OECT)-based sensor named a bioristor has been developed and applied in plant stems for the continuous, precise and real-time monitoring of the changes occurring in the plant sap composition, during growth and development and upon drought stress and environmental changes [13,50,55,56] in controlled conditions. Its application allowed for the early warning of drought stress [13] and for dynamically tracing the saline stress response in giant cane [55]. The possibility of monitoring the plant’s health status and early phases of drought stress directly from the stem can consistently improve water use efficiency in agriculture and increase crop production sustainability. High reproducibility and stability in measurements between tomato plant replicates have been reported [13,57]; moreover, the bioristor’s scalability for use in diverse crop species has been reported [55,58].

Tomato (*Solanum lycopersicum* L.) is one of the most cultivated vegetables in the world, with about 189 million tons cultivated in 2021 according to the Food and Agriculture Organization of the United Nations (FAOSTAT, 2023; http://www.fao.org/faostat/en/#data/QC; accessed on 6 July 2023) and with a total addressable market (TAM) valued at USD 181.74 billion in 2022 [59]. During tomato field growth, several abiotic and biotic stresses occur and strongly affect final yield and quality [60,61].

In tomato, drought stress significantly affects yield [62]. The tomato plant is sensitive to lack of water during reproduction, especially during flowering and fruit growth [63]. Novel approaches are needed to reach the goal of more sustainable agriculture that has lower water requirements and promotes resistance to biotic and abiotic stress [63]. 

So far, image-based remote and proximal sensing platforms have been individually applied to monitor the drought stress response of tomato. Examples are UAVs equipped with multispectral, hyperspectral [64] and thermal [65] sensors. Only recently, an innovative in vivo sensor named a bioristor was also used to monitor tomatoes in open fields [57] to improve irrigation efficiency.

The objectives of the present study were (a) to analyze the strength of a multiscale approach for PA, (b) to determine the relationships between vegetation indices and drought stress and (c) to demonstrate the effectiveness of bioristor in monitoring the water needs of tomato plants in an open field at the micro-scale level.

To this end, the performance of multispectral and thermal sensors mounted on a UAV will be compared with that of a low-cost RGB sensor and with that of a bioristor. 

The results are discussed in terms of the efficiency of the multiscale approach and the correlation between the acquired indices and the physiological or environmental traits investigated.

## 2. Results

### 2.1. The Macro Scale: UAV Multispectral Remote Imaging

#### Acquisition of Multispectral and Thermal Vegetation Indices

**Figure 1:**
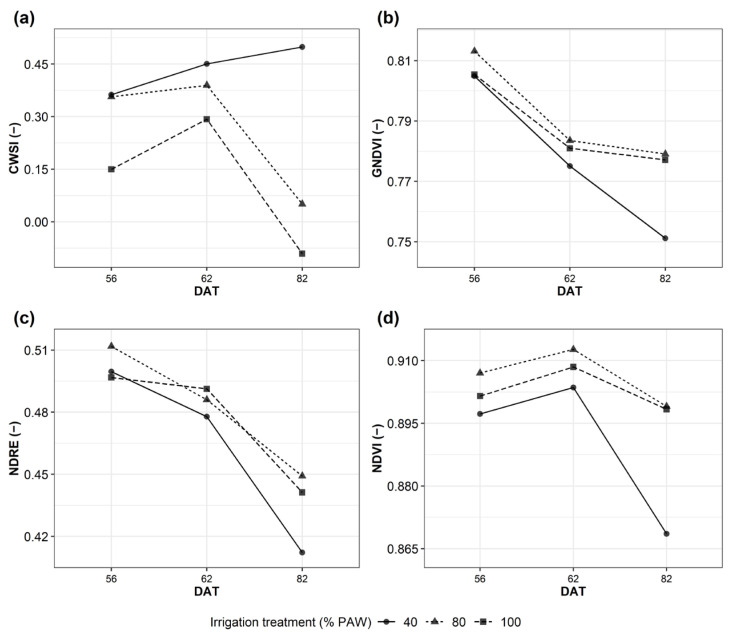
Multispectral and thermal indices. CWSI (**a**), Crop Water Stress Index, GNDVI (**b**), Green Normalized Difference Vegetation Index, NDRE (**c**), Normalized Difference Red Edge Index, NDVI (**d**), Normalized Difference Vegetation Index. DAT, days after transplant; PAW, plant available water.

CWSI values increased at 56 days after transplant (DAT) up to 82 DAT for 40% PAW, while a decrease in CWSI values was observed at 82 DAT for the 80% and 100% PAW (Figure 1a). Regarding the multispectral VIs, GNDVI and NDRE values decreased from 56 DAT to 82 DAT (Figure 1b,c), while NDVI value reached a maximum value at 62 DAT (Figure 1d). The largest differences between the 40% PAW irrigation treatment and the 80% and 100% PAW were observed at 82 DAT for GNDVI, NDRE, NDVI and CWSI indices (Figure 1).

### 2.2. Medium Scale: RGB Imaging (Proximal)

**Figure 2:**
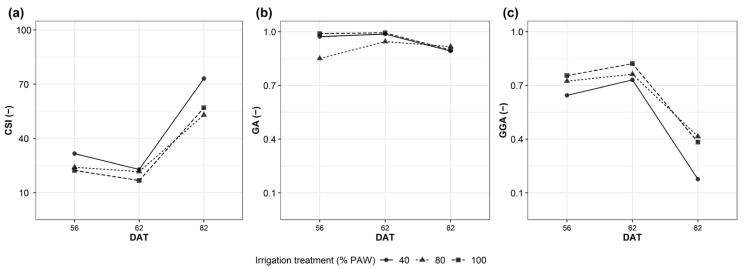
RGB-based vegetation indices (VIs). (**a**) CSI; (**b**) GA index, %; (**c**) GGA. Water conditions analyzed: 100%, 80% and 40% PAW.

During the field trial, plots corresponding to 100% and 80% PAW did not show significant differences in the GA index (Figure 2b). On the contrary, 40% PAW showed a significant difference in GA for the entire set of measures (for 17 days). During the fruit-set development (from day 32), 40% PAW showed a 4% GA reduction compared to 100% PAW (*p* ≤ 0.001), reaching the minimum canopy at the ripening stage (day 62, 19% GA reduction compared with 100% PAW (*p* ≤ 0.001, Figure 2b). 

GGA showed a rapid decrease in the 100% PAW plots (Figure 2c, Supplementary Figure S2c) because of the smaller fraction of green pixels captured with canopy images and the rapid increases in the red pixels in the 100% PAW plots during fruit set and ripening.

The CSI was also calculated based on GA and GGA. It supported the hypothesis of a faster ripening behavior of the 100% PAW plots and a strong reduction of plant development and rapid senescence in the 40% PAW plots (Figure 2a). 

### 2.3. Bioristor, the Micro-Scale Approach for In Vivo Plant (Ground) Monitoring

**Figure 3:**
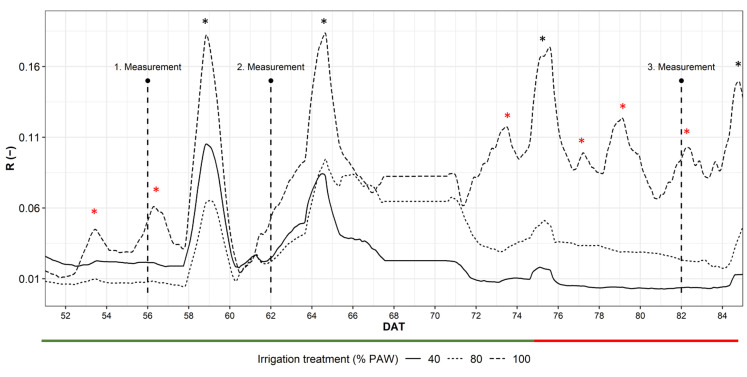
Plot of the daily smooth average of the sensor response R; 100%, 80% and 40% irrigation treatment (PAW). Black asterisks indicate rainy events; red asterisks indicate abundant irrigations; dashed blocks indicate the R windows considered for the correlation with yield. Tomato maturation stages are indicated with colored bars: green, turning stage; red, fruit ripening. UAV measurements: 1, 56 DAT; 2, 62 DAT, 3, 82 DAT. DAT, days after transplant; PAW, plant available water. Black asterisks indicate rainy events, red asterisks abundant irrigations.

A bioristor was used to detect the changes occurring in the plant physiology under water shortage at the micro-scale level. During the experiments, tomato growth from transplant to harvest was monitored in real time and continuously for 60 days, giving a complex but interesting picture of the changes occurring in the plant during growth and development under natural cultivation conditions (Figure 3).

**Figure 4:**
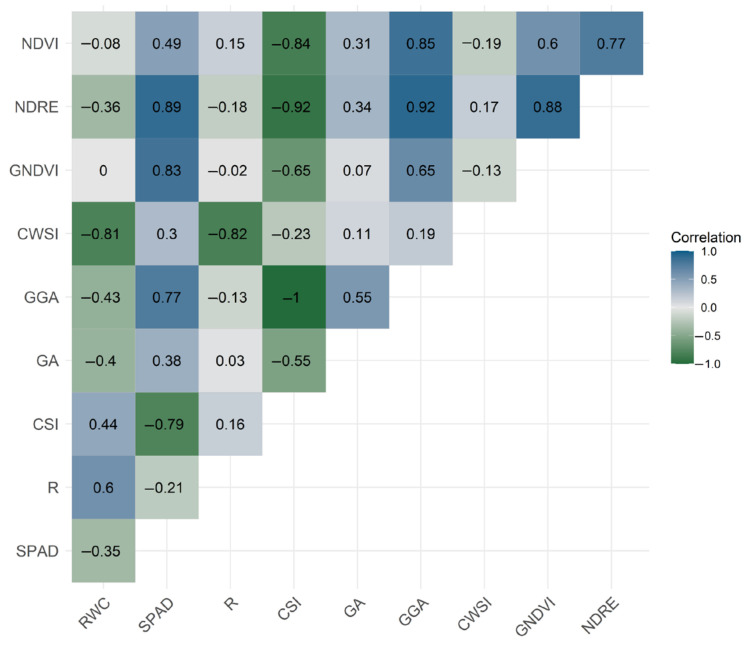
Correlation matrix. RWC, relative water content; SPAD; R, bioristor sensor response; CSI, Crop Stress Index; GA, Green Area; GGA, Greener Area; CWSI, Crop Water Stress Index; NDRE, Normalized Difference Red Edge Index.

An increase in R was observed during rainy events as proportional to the intensity of the rain (Figure 4) but also during the irrigation sessions.

The R trend showed no significant changes in the overall plant health status, indicated by the changes in the slope of R during time up to 60 DAT (Figure 3). Differential irrigation was applied at 43 DAT, but the occurrence of several rainy events hampered a real differentiation of the overall water available in the soil as an effect of the different irrigation. From 65 to 90 DAT, the R trend showed an appreciable and significant difference within the treatments (*p* ≤ 0.001), where the R of the 40% PAW plots rapidly dropped from 65 DAT to the end of the experiment, while 100% and 40% were well separated according to the given amount of water only from 72 DAT (Figure 3).

### 2.4. Physiological Plant Health Traits Analyzed and Yield Components

Manual physiological measurements were also performed to validate the sensor-based phenotyping at 43, 47, 50, 56, 62, 66, 74, 81 and 89 DAT. 

**Figure 5:**
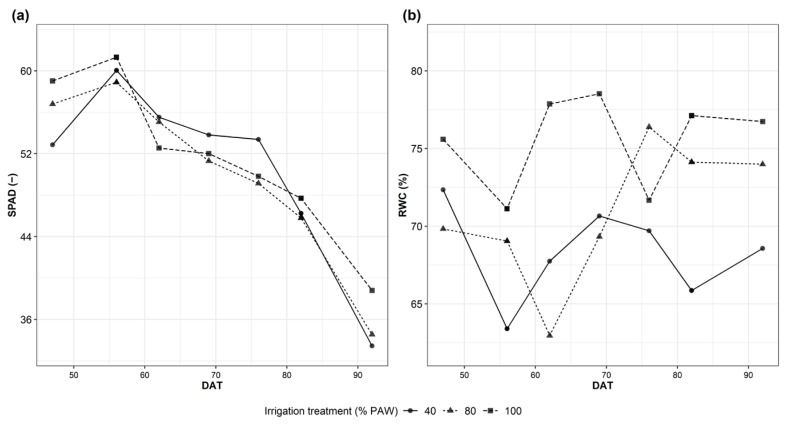
Physiological measurements. Plants were tested at 47, 56, 62, 69, 76, 82 and 92 days after transplant (DAT). (**a**) Relative water content (RWC); (**b**) relative SPAD unit for measuring chlorophyll content.

The relative water content (RWC) and the SPAD values were evaluated and reported for all treatments to acquire a direct measurement of the plant status (Figure 5). 

**Figure 6:**
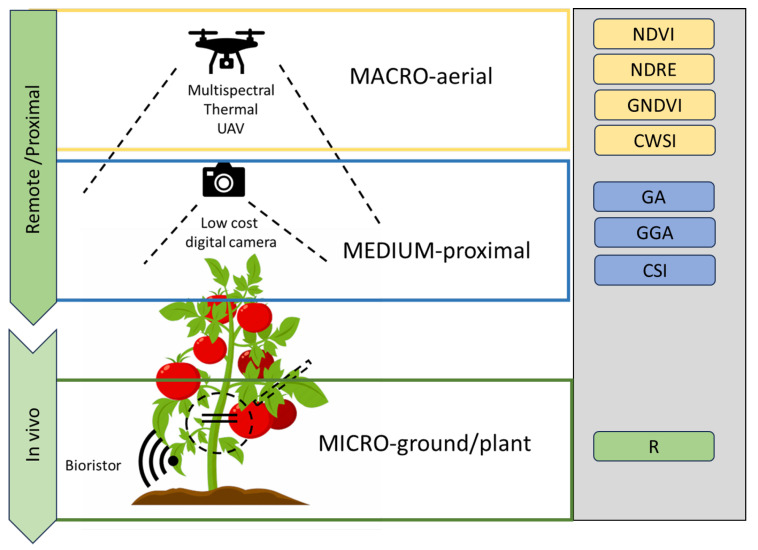
Multiscale monitoring approach used in tomato field cultivation. Macro aerial traits analyzed: Normalized Difference Vegetation Index, NDVI; Normalized Difference Red Edge Index, NDRE; Green Normalized Difference Vegetation Index, GNDVI; Crop Water Stress Index, CWSI; Green Area, GA; Greener Area, GGA; Crop Senescence Index, CSI; Sensor Response Index, R (see Supplementary Table S2 for details).

A significant difference, mainly between the 100 and 40% treatments, was observed for RWC and SPAD on day 56 (Figure 5). Interestingly, the RWC data and the bioristor data are not in agreement in the initial days of phase 3 (Figure 4 and Figure 6).

The physiological data confirm the similarity of the health status occurring at 100% and 80% PAW and highlight that at 20% PAW is the most affected by drought stress.

SPAD values do not show any significant difference for the entire length of the experiment (Figure 5a).

The total yield was 74.9 t ha^−1^, 94.3 t ha^−1^ and 100.2 t ha^−1^ for the three different irrigation treatments (Table 1, 40%, 80% and 100% PAW), respectively. Only the 40% PAW treatment showed a significant reduction in the total production as well as marketable production due to a significant increase in the yield of rotten fruits, because of the severe drought stress (Table 1). 

Based on the data collected, a correlation analysis was performed to verify the relationship between RGB, multispectral, thermal, bioristor and physiological indices (Figure 5, Supplementary Figures S2–S4). 

RWC was highly correlated with water-related indices like CWSI and R, negatively and positively, respectively, and correlated, to a lower degree, with CSI (positively) and GA and GGA (negatively).

The SPAD index showed a high correlation with the RGB index CSI (negatively), GGA, GNDVI and NDRE and was moderately correlated with NDVI but had an extremely low correlation with R and CWSI.

NDVI showed a good correlation with GGA, GNDVI and NDRE and a negative high correlation with CSI. A medium correlation was observed with the SPAD index. 

Similarly, NDRE was negatively correlated with GGA, GNDVI and SPAD and was highly anticorrelated with CSI.

The CWSI showed a high negative correlation with R and RWC.

CSI showed anticorrelation with almost all the analyzed indices, SPAD, NDVI, NDRE, GNDI and GA.

GGA showed a high correlation with SPAD, GA, NDVI and NDRE.

The main findings reside in the analysis of the R correlation with known indices. It showed a high peculiar correlation only with CWSI (r = −0.82) and a medium correlation with RWC (r = 0.51).

## 3. Materials and Methods

### 3.1. The Approach

To verify the multiscale approach applied in this research paper, a range of sensors covering the macro, medium and micro scales were applied, as summarized in Figure 6. A UAV was applied as a remote platform, RGB low-cost imaging was applied as a proximal medium-scale sensor and a bioristor was applied for in vivo micro-scale monitoring.

### 3.2. Field Trial Description and Stress Conditions

A field trial was carried out in 2019 at Podere Stuard, in Parma (60 m a.s.l., 44°48′29.88′′ N 10°16′29.074′′ E). The tomato cv. Heinz was chosen for the field trial. A randomized block approach was adopted using three plots divided into three rows for each water treatment. The middle row of one plot for each water regime was monitored with a bioristor by measuring 5 plants. The irrigation treatments expressed as plant available water (PAW) irrigation treatments were established based on the irrigation advice defined by Irriframe (https://www.irriframe.it/Irriframe (accessed on 8 September 2023) as 100%, 80% and 40% PAW.

The full list of field management and main operations including watering, fertilization and soil tillage is reported in Supplementary Table S1.

### 3.3. Environmental Conditions: Soil Humidity Sensors and Meteorological Data

Data on rainfall volume (mm) and relative humidity (RH%) at 2 m above the ground were collected by the agrometeorological station of the ARPAE network (https://simc.arpae.it/dext3r/, (accessed on 20 October 2023); Supplementary Figure S1).

### 3.4. Bioristor Preparation and Implementation

The bioristor was prepared according to Janni et al., 2019. In brief, two textile fibers were treated by soaking them for 5 min in aqueous poly(3,4-ethylenedioxythiophene) doped with polystyrene sulfonate (Clevios PH1000, Starck GmbH, Munich, Germany), and dodecyl benzene sulfonic acid (2% *v*/*v*) was added. The fibers were then baked at 130 °C for 30 min. The whole process, from deposition to heat treatment, was repeated 3 times to complete the preparation. Then, a treatment with concentrated sulfuric acid (95%) was performed for 20 min to increase the crystallinity of the polymer and, therefore, its electrical properties, as well as its duration over time. Before functionalization, each thread was cleaned by plasma oxygen cleaner treatment (Femto, Diener electronic, Ebhausen, Germany) to increase its wettability and to facilitate the adhesion of the aqueous conductive polymer solution. 

**Figure 7:**
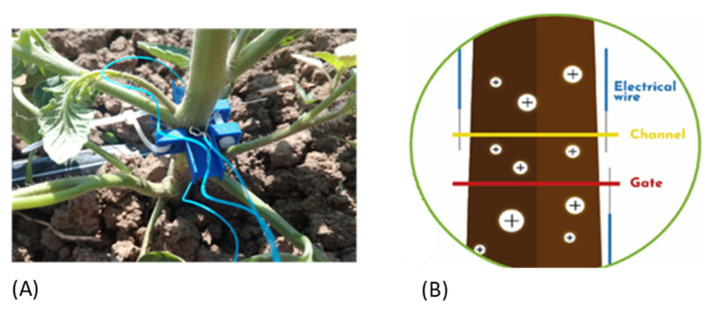
Bioristor monitoring. (**A**) Bioristor installed in the plant stem, (**B**) bioristor working principle.

One bioristor was inserted in the plant stem of 5 plants at the 5-leaf stage by opening a hole using a needle. Five replicas for each water regime were analyzed. The treated fiber was completely inserted into the plant stem. The fiber was connected on each end to a metal wire with silver paste to stabilize the connections, forming the “source” and “drain” electrodes. The transistor device was completed by inserting a second fiber functionalized as a gate electrode (Figure 7A,B). A constant voltage (Vds = −0.1 V) was applied across the main transistor channel, along with a positive voltage at the gate (Vg = 0.5 V) which led to a decrease in channel conductivity due to the cations pushed from the electrolyte into the channel; the resulting current (Ids) was monitored continuously (Figure 2b). The sensor response (R) was acquired and reported in both experiments. It is proportional to the cations present in the electrolyte and is given by the expression |Ids − Ids0|/Ids0, where Ids0 represents the current across the channel when Vg = 0 V.

The bioristor elements were connected to a NI USB−6343 multifunction I/O device (National Instruments, Austin, TX, USA), which is a multi-channel digital–analog converter, connected to a PC where current data were processed using home-made software and then saved in the cloud.

### 3.5. RGB-Based Imaging

Vegetation indices derived from RGB images were evaluated for each plot at ground level as reported by Gracia-Romero et al., 2019 [28], with slight modifications.

One picture per plot was taken while a cell phone was held at 80 cm above the plant canopy. To facilitate the procedure, the camera was attached to a monopod to adjust and stabilize the distance between the camera and the top of the canopy. Images were saved in JPEG format at a resolution of 4608 × 3072 pixels. Two plots for each water regime were investigated using RGB imaging.

To calculate the vegetation indices, the RGB images were processed with MosaicTool (https://www.gitlab.com/sckefauver/MosaicTool, University of Barcelona, Barcelona, Spain) integrated as a plugin for FIJI (Fiji is Just ImageJ; https://www.fiji.sc/Fiji/) [41] that enables the extraction of RGB indices in relation to different color properties of potential interest [44]. Derived from the hue–intensity–saturation (HIS) color space, average values from all the pixels of the image were determined for hue, referring to the color tint; saturation, an indication of how much the pure color is diluted with white color; and intensity, as an achromatic measurement of the reflected light. In addition, the portion of pixels with hue classified as green was determined with the Green Area (GA) and Greener Area (GGA) indices. 

GA is the percentage of pixels in the image with a hue range from 60° to 180°, including yellow to bluish-green color values. 

GGA is more restrictive, because it reduces the range from 80° to 180°, thus excluding the yellowish-green tones. Both indices are also used for the formulation of the Crop Senescence Index (CSI) [66], which provides a scaled ratio between yellow and green pixels to assess the percentage of senescent vegetation. From the CIELab and the CIELuv color space models (recommended by the International Commission on Illumination (CIE) for improved color chromaticity compared to the HIS color space), dimension L* represents lightness and is very similar to intensity from the HIS color space, whereas a* and u* represent the red–green spectrum of chromaticity, and b* and v* represent the yellow–blue color spectrum. 

### 3.6. UAV Multispectral and Thermal Image-Based Indices

Unmanned aerial vehicle (UAV) multispectral and thermal images were collected using a DJI Matrice 210 RTK Quadcopter (SZ DJI Technology Co., Shenzhen, Guangzhou, China) for three field campaigns (Supplementary Table S1) performed at 56 days after transplant (DAT), 62 DAT and 82 DAT on the entire field. The UAV was equipped with a DJI FLIR Zenmuse XT2 high-resolution radiometric thermal camera and a MicaSense RedEdge-Mx multispectral camera (MicaSense, Seattle, WA, USA) which acquired five multispectral images [35]. Flights were performed in clear sky conditions, and the flight altitude was 30 m above ground level (AGL). The forward and lateral overlaps were set at 80% and 75% of the images, respectively. A light sensor mounted at the top of the UAV and a reflectance panel provided by MicaSense were used for the radiometric calibration of the multispectral images. The radiometric calibration and orthomosaic generation (both for multispectral and thermal images) were performed using the Pix4D mapper (Pix4D, S.A., Lausanne, Switzerland). Five vegetation indices (VIs), such as Green Normalized Difference Vegetation Index (GNDVI, [67]), Normalized Difference Red Edge Index (NDRE, [68]) and Normalized Difference Vegetation Index (NDVI, [69]), were calculated using the following equations:

(1) $$GNDVI=\frac{R_{nir}-R_{green}}{R_{nir}+R_{green}}$$

(2) $$NDRE=\frac{R_{nir}-R_{rededge}}{R_{nir}+R_{rededge}}$$

(3) $$NDVI=\frac{R_{nir}-R_{red}}{R_{nir}+R_{red}}$$

where $R_{green}$, $R_{red}$, $R_{rededge}$ and $R_{nir}$ are reflectance values of vegetation in the green, red, red edge and near-infrared bands extracted from the multispectral orthomosaics.

Crop Water Stress Index (CWSI) was calculated, according to the methodology proposed by Idso et al. (1982) [70], using the following equations:

(4) $$CWSI=\frac{\left(T_{c}-T_{a}\right)-{\left(T_{c}-T_{a}\right)}_{LL}}{{\left(T_{c}-T_{a}\right)}_{UL}-{\left(T_{c}-T_{a}\right)}_{LL}}$$

(5) $${\left(T_{c}-T_{a}\right)}_{LL}=a+b\times VPD$$

(6) $${\left(T_{c}-T_{a}\right)}_{UL}=a+b\times VPG$$

where $\left(T_{c}-T_{a}\right)$ is the difference between $T_{c}$, the canopy temperature extracted from thermal orthomosaics, and $T_{a}$, the air temperature; ${\left(T_{c}-T_{a}\right)}_{LL}$ and ${\left(T_{c}-T_{a}\right)}_{UL}$ are the lower and upper limits of the canopy temperature difference calculated using VPD (kPa) and VPG (kPa), which are the vapor pressure deficit (VPD) and vapor pressure gradient (VPG) calculated as the difference between the air-saturated water vapor pressure at temperature $T_{a}$ and the air-saturated water vapor pressure at temperature $T_{a}$ + a; a is the intercept and b is the slope of the linear regression. 

The averages of the VIs and CWSI for each experimental plot were extracted from pure vegetation pixels which were classified by applying a k-means clustering algorithm on multispectral and thermal orthomosaics for segmenting vegetation from the soil.

A full list of the variables considered is presented in Supplementary Table S2.

### 3.7. Physiological Measurements: Water Status and Fluorescence

Five plants for each treatment were analyzed for the leaves’ relative water content (RWC) as reported by Janni et al., 2019 [13]. Chlorophyll content measurements were performed by using a SPAD 502 m (Konica Minolta, Ramsey, NJ, USA) on three expanded leaves; the relative SPAD value was recorded. 

### 3.8. Yield Assessment

Yield components were recorded at the end of the experiment for all plants for each water regime: total production (t ha^−1^), commercial yield (t ha^−1^), unripe product (t ha^−1^) and rotten product (t ha^−1^).

### 3.9. Data Analysis and Statistics

The R value was analyzed with MATLAB (https://uk.mathworks.com/) and Microsoft Excel 2016 to smooth day/night oscillations and scaled through a min–max normalization (0.1 range). Bioristor data were statistically analyzed by applying analysis of variance (ANOVA) in MatLab 2014a (8.3.0.532). Mean, standard deviation and standard error were calculated.

## 4. Discussion

Plant stress detection is considered one of the most critical areas for the improvement of crop yields in the compelling worldwide scenario of ongoing climate change [25,71]. Agricultural equipment has become more efficient, reliable, and precise thanks to automation and the increased use of robotics and sensors for plant monitoring.

The multiscale approach for plant monitoring presented in this work can significantly improve the detection of water stress at the field level [72,73,74,75]. Remote sensing methods and image spectral analysis are applied in precision agriculture (PA), can analyze soil state and vegetation health from a distance and are image-based [76]. Moreover, RGB or color cameras are the most basic vision-based sensors. Color data may be used to determine parameters such as texture and geometrical characteristics, which are important in agricultural applications [76]. Lastly, proximal sensors can measure soil qualities directly or indirectly and are close to, or even in contact with, the ground. The use of such advanced sensors and tools can provide farmers with valuable insights into crop growth and yield [77].

However, the complete lack of sensors that enable dynamic and continuous monitoring of plant water stress was observed [29,47]. 

In this study, an in vivo biosensor named a bioristor was coupled with remote and proximal sensing techniques as a tool for precision agriculture, and it was demonstrated that the methodologies presented were capable of monitoring tomato plants’ response to water conditions. 

Our results showed that the highest correlations observed were between photosynthesis and chlorophyll-related traits (SPAD and related indices with RGB indices (GGA and CSI)) and between multispectral vegetation indices (NDVI, NDRE) and chlorophyll-related traits (SPAD), as previously reported [47,78]. The high correlation observed between chlorophyll-related indices such as GNDVI, NDRE and SPAD is in line with previously reported data [79,80].

In disagreement with reported data for grapes [81,82], NDVI and GNDVI do not correlate with transpiration-related traits such as RWC and CWSI [70,83]. Also, the bioristor R index does not show a correlation with NDVI.

Moreover, the NDRE, NDVI, GGA and CSI trends with the time of measurement confirm their ability to trace the course of the first slow and then fast maturation of tomato plants and the possible use of these indices as integrative measurements of the overall amount and quality of photosynthetic material in plants or the combined effects of leaf chlorophyll content, canopy leaf area or architecture [78].

R, on the contrary, showed a specific and high correlation with water-related indices that specifically trace the effects of drought stress on plants (RWC, CWSI). No correlation was observed with CSI and NDVI, confirming its high specificity in monitoring changes in values of ions flowing in the transpiration stream and thus monitoring the plant water status. 

Of particular interest is the high negative correlation observed between R and CWSI (r = −0.82), a measure of the relative transpiration rate occurring in the plant and described as more accurate in determining the soil and plant water status [84].

A strong relationship between vegetation indices and VPD was reported [85,86,87]. The correlation between VPD and transpiration-related and water-use-related indices like WUE, CWSI and R is extremely high [56,88,89,90,91]. Also, NDVI has been reported to be influenced by VPD [92,93]. 

These data support the negative correlation between R and VPD as reported by Vurro et al., 2019 [56], further demonstrating the bioristor’s ability to detect physiological changes caused by transpiration.

Under low VPD and high CWSI conditions, a high R was observed, indicating the efficacy of bioristor in detecting the occurrence of transpiration during plant growth development and under water shortage.

Due to the ability of the bioristor to monitor the plant water status continuously and in real time, this study further supports its use for precision irrigation; the bioristor is as accurate as CWSI but allows the dynamic and continuous tracing of the plant water status. 

In addition, when compared with the total yield, R showed a good correlation (r = 0.82), confirming the link between water use efficiency and yield.

**Figure 8:**
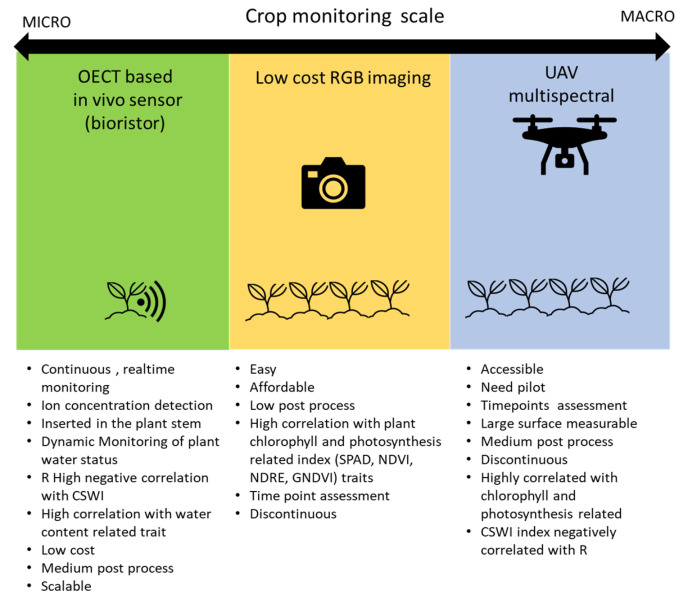
Advantages and disadvantages of the techniques used in the present work, from macro- to micro-scale phenotyping.

In this study, we validated the use of multiscale vegetation indices developed from UAV, low-cost proximal RGB and in vivo monitoring methods to predict tomato water needs and to determine local irrigation requirements [94]. An evaluation of each scale of monitoring is reported in Figure 8.

## 5. Conclusions

This work provides an overview of three phenotyping approaches for evaluating drought-related functional traits at various observational scales. First, the bioristor, presented as a micro-scale methodology, enabled the continuous monitoring of the plant water status; the bioristor’s ion concentration measurements in the transpiration stream are used as a direct estimation of plant water use. 

Having real-time information about the plant’s status helps to identify when it starts responding to stress. However, the application of this methodology may be limited under field conditions, as a high number of sensors would be needed to obtain a precise representation. On the other hand, remote sensing methodologies based on the calculation of vegetation indices at the canopy level, presented at medium and macro scales, have also been reported as good indicators of drought response. Unlike the micro-scale strategy, proximal remote sensing streamlines the selection process by reducing the time required to assess extensive experimental fields. In return, drought response is evaluated indirectly through estimations of green biomass using RGB and multispectral indices which are highly correlated to the measures of chlorophyll content, as well as by measuring transpiration rates through canopy temperature assessment which reported better associations with the water-content-related traits measured by the bioristor. Proximal remote sensing enhances throughput capacity but may sacrifice precision in estimating the response and the ability to determine the onset of stress. The main difference between the medium and macro scales is that UAV technology allows for the assessment of larger populations more quickly, although the distance between the target and the sensor can affect image resolution compared to ground-level evaluations. Finally, conventional RGB cameras used at the medium scale represent a cost-effective alternative to the more expensive methodologies used at the macro level. Despite these differences, measurements at both levels performed similarly when assessing tomato plants.

In summary, the combination of technologies described provides a comprehensive understanding of plants, their physiological functions, and their interaction with the environment.

## Figures and Tables

**Table 1:** Yield traits analyzed. (a) Total yield and (b) marketable fruit expressed in t ha^−1^. Different letters indicate significant differences between irrigation treatments (HSD Tukey test, *p* < 0.05).

%PAW	Marketable (t ha^−1^)		Unripen (t ha^−1^)	Rotten (t ha^−1^)		Total Yield (t ha^−1^)		
100%	73	a	23.2	a	3.9	b	100.2	a
80%	68.2	a	19.6	a	6.4	b	94.3	a
40%	50.4	b	11.4	b	13.1	a	74.9	b
Mean	63.9		18.1		7.8		89.8	
CV (%)	8.86		17.49		32.82		8.25	
Significance	**		**		**		**	

Significance: (**) *p* = 0.01.

## Data Availability

The data presented in this study are available on request from the corresponding author.

## Supplementary Materials

The following supporting information can be downloaded at: https://www.mdpi.com/article/10.3390/plants12223851/s1, Table S1: Field operations performed in 2019; Table S2: Vegetation and bioristor-based indices; Figure S1: Environmental measurements; Figure S2: Correlation plot between multiscale-acquired indices; Figure S3: Correlation plot between the SPAD index and other indices; Figure S4: Correlation plot between the RWC and other indices.

## References

[1] Roper J.M., Garcia J.F., Tsutsui H. (2021). Emerging Technologies for Monitoring Plant Health in Vivo. ACS Omega.

[2] Janni M., Pieruschka R. (2022). Plant Phenotyping for a Sustainable Future. J. Exp. Bot..

[3] Muhie S.H. (2022). Novel Approaches and Practices to Sustainable Agriculture. J. Agric. Food Res..

[4] Dhanaraju M., Chenniappan P., Ramalingam K., Pazhanivelan S., Kaliaperumal R. (2022). Smart Farming: Internet of Things (iot)-Based Sustainable Agriculture. Agriculture.

[5] Yuan M., Zheng N., Yang Y., Liu C. (2023). Robust Optimization for Sustainable Agricultural Management of the Water-Land-Food Nexus under Uncertainty. J. Clean. Prod..

[6] Seleiman M.F., Hafez E.M., Awaad H., Abu-hashim M., Negm A. (2021). Optimizing Inputs Management for Sustainable Agricultural Development. Mitigating Environmental Stresses for Agricultural Sustainability in Egypt.

[7] White J.C., Gardea-Torresdey J. (2021). Nanoscale Agrochemicals for Crop Health: A Key Line of Attack in the Battle for Global Food Security. Environ. Sci. Technol..

[8] Verma K.K., Song X.-P., Joshi A., Tian D.-D., Rajput V.D., Singh M., Arora J., Minkina T., Li Y.-R. (2022). Recent Trends in Nano-Fertilizers for Sustainable Agriculture under Climate Change for Global Food Security. Nanomaterials.

[9] Shalaby T.A., Bayoumi Y., Eid Y., Elbasiouny H., Elbehiry F., Prokisch J., El-Ramady H., Ling W. (2022). Can Nanofertilizers Mitigate Multiple Environmental Stresses for Higher Crop Productivity?. Sustainability.

[10] Rajput V.D., Singh A., Minkina T., Rawat S., Mandzhieva S., Sushkova S., Shuvaeva V., Nazarenko O., Rajput P., Komariah (2021). Nano-Enabled Products: Challenges and Opportunities for Sustainable Agriculture. Plants.

[11] Gupta A., Rayeen F., Mishra R., Tripathi M., Pathak N. (2023). Nanotechnology Applications in Sustainable Agriculture: An Emerging Eco-Friendly Approach. Plant Nano Biol..

[12] Nongbet A., Mishra A.K., Mohanta Y.K., Mahanta S., Ray M.K., Khan M., Baek K.-H., Chakrabartty I. (2022). Nanofertilizers: A Smart and Sustainable Attribute to Modern Agriculture. Plants.

[13] Janni M., Coppede N., Bettelli M., Briglia N., Petrozza A., Summerer S., Vurro F., Danzi D., Cellini F., Marmiroli N. (2019). In Vivo Phenotyping for the Early Detection of Drought Stress in Tomato. Plant Phenomics.

[14] Marios S., Georgiou J. Precision Agriculture: Challenges in Sensors and Electronics for Real-Time Soil and Plant Monitoring. Proceedings of the 2017 IEEE Biomedical Circuits and Systems Conference (biocas).

[15] Rovira-Más F., Saiz-Rubio V., Cuenca-Cuenca A. (2021). Sensing Architecture for Terrestrial Crop Monitoring: Harvesting Data as an Asset. Sensors.

[16] Padilla-Medina J.A., Contreras-Medina L.M., Gavilán M.U., Millan-Almaraz J.R., Alvaro J.E. (2019). Sensors in Precision Agriculture for the Monitoring of Plant Development and Improvement of Food Production. J. Sens..

[17] ANSA Water Crisis Threatens 18% of Italy’s GDP. https://www.ansa.it/english/news/general_news/2023/03/22/water-crisis-threatens-18-of-italys-gdp-report_d074853f-b02f-42a2-b677-81a4d5dc8a61.html.

[18] Araus J.L., Cairns J.E. (2014). Field High-Throughput Phenotyping: The New Crop Breeding Frontier. Trends Plant Sci..

[19] Araus J.L., Kefauver S.C., Vergara-Díaz O., Gracia-Romero A., Rezzouk F.Z., Segarra J., Buchaillot M.L., Chang-Espino M., Vatter T., Sanchez-Bragado R. (2022). Crop Phenotyping in a Context of Global Change: What to Measure and How to Do It. J. Integr. Plant. Biol..

[20] Atkinson J.A., Jackson R.J., Bentley A.R., Ober E., Wells D.M. (2018). Field Phenotyping for the Future. Annual Plant Reviews Online.

[21] Chawade A., van Ham J., Blomquist H., Bagge O., Alexandersson E., Ortiz R. (2019). High-Throughput Field-Phenotyping Tools for Plant Breeding and Precision Agriculture. Agronomy.

[22] De Swaef T., Maes W., Aper J., Baert J., Cougnon M., Reheul D., Steppe K., Roldàn-Ruiz I., Lootens P. (2021). Applying RGB- and Thermal-Based Vegetation Indices from uavs for High-Throughput Field Phenotyping of Drought Tolerance in Forage Grasses. Remote Sens..

[23] Kefauver S.C., Vicente R., Vergara-Díaz O., Fernandez-Gallego J.A., Kerfal S., Lopez A., Melichar J.P.E., Molins S., D M., Araus J.L. (2017). Comparative UAV and Field Phenotyping to Assess Yield and Nitrogen Use Efficiency in Hybrid and Conventional Barley. Front. Plant Sci..

[24] Li D., Quan C., Song Z., Li X., Yu G., Li C., Muhammad A. (2021). High-Throughput Plant Phenotyping Platform (HT3P) as a Novel Tool for Estimating Agronomic Traits From the Lab to the Field. Front. Bioeng. Biotechnol..

[25] Morisse M., Wells D.M., Millet E.J., Lillemo M., Fahrner S., Cellini F., Lootens P., Muller O., Herrera J.M., Bentley A.R. (2022). A European Perspective on Opportunities and Demands for Field-Based Crop Phenotyping. Field Crops Res..

[26] Wu S., Wen W., Wang Y., Fan J., Wang C., Gou W., Guo X. (2020). MVS-Pheno: A Portable and Low-Cost Phenotyping Platform for Maize Shoots Using Multiview Stereo 3D Reconstruction. Plant Phenomics.

[27] Tao H., Xu S., Tian Y., Li Z., Ge Y., Zhang J., Wang Y., Zhou G., Deng X., Zhang Z. (2022). Proximal and Remote Sensing in Plant Phenomics: 20 Years of Progress, Challenges, and Perspectives. Plant Commun..

[28] Gracia-Romero A., Kefauver S.C., Fernandez-Gallego J.A., Vergara-Díaz O., Nieto-Taladriz M.T., Araus J.L. (2019). UAV and Ground Image-Based Phenotyping: A Proof of Concept with Durum Wheat. Remote Sens..

[29] Harfouche A.L., Jacobson D.A., Kainer D., Romero J.C., Harfouche A.H., Scarascia Mugnozza G., Moshelion M., Tuskan G.A., Keurentjes J.J.B., Altman A. (2019). Accelerating Climate Resilient Plant Breeding by Applying Next-Generation Artificial Intelligence. Trends Biotechnol..

[30] Maes W.H., Steppe K. (2019). Perspectives for Remote Sensing with Unmanned Aerial Vehicles in Precision Agriculture. Trends Plant Sci..

[31] Matias F.I., Caraza-Harter M.V., Endelman J.B. (2020). fieldimager: An R Package to Analyze Orthomosaic Images from Agricultural Field Trials. Plant Phenome J..

[32] Morales N., Kaczmar N.S., Santantonio N., Gore M.A., Mueller L.A., Robbins K.R. (2020). imagebreed: Open-Access Plant Breeding Web–Database for Image-Based Phenotyping. Plant Phenome J..

[33] Bonfante A. (2019). LCIS DSS—An Irrigation Supporting System for Water Use Efficiency Improvement in Precision Agriculture–A Maize Case Study. Agric. Syst..

[34] Polinova M., Salinas K., Bonfante A., Brook A. (2019). Irrigation Optimization Under a Limited Water Supply by the Integration of Modern Approaches into Traditional Water Management on the Cotton Fields. Remote Sens..

[35] Impollonia G., Croci M., Ferrarini A., Brook J., Martani E., Blandinières H., Marcone A., Awty-Carroll D., Ashman C., Kam J. (2022). UAV Remote Sensing for High-Throughput Phenotyping and for Yield Prediction of Miscanthus by Machine Learning Techniques. Remote Sens..

[36] Impollonia G., Croci M., Martani E., Ferrarini A., Kam J., Trindade L.M., Clifton-Brown J., Amaducci S. (2022). Moisture Content Estimation and Senescence Phenotyping of Novel Miscanthus Hybrids Combining UAV-Based Remote Sensing and Machine Learning. GCB Bioenergy.

[37] Impollonia G., Croci M., Blandinières H., Marcone A., Amaducci S. (2022). Comparison of PROSAIL Model Inversion Methods for Estimating Leaf Chlorophyll Content and LAI Using UAV Imagery for Hemp Phenotyping. Remote Sens..

[38] Antonucci G., Impollonia G., Croci M., Potenza E., Marcone A., Amaducci S. (2023). Evaluating Biostimulants via High-Throughput Field Phenotyping: Biophysical Traits Retrieval through PROSAIL Inversion. Smart Agric. Technol..

[39] Casadesús J., Villegas D. (2014). Conventional Digital Cameras as a Tool for Assessing Leaf Area Index and Biomass for Cereal Breeding. J. Integr. Plant Biol..

[40] Fernandez-Gallego J.A., Kefauver S.C., Vatter T., Aparicio Gutiérrez N., Nieto-Taladriz M.T., Araus J.L. (2019). Low-Cost Assessment of Grain Yield in Durum Wheat Using RGB Images. Eur. J. Agron..

[41] Gracia-Romero A., Kefauver S.C., Vergara-Díaz O., Zaman-Allah M.A., Prasanna B.M., Cairns J.E., Araus J.L. (2017). Comparative Performance of Ground vs. Aerially Assessed RGB and Multispectral Indices for Early-Growth Evaluation of Maize Performance under Phosphorus Fertilization. Front. Plant Sci..

[42] Gozdowski D., Stępień M., Panek E., Varghese J., Bodecka E., Rozbicki J., Samborski S. (2020). Comparison of Winter Wheat NDVI Data Derived from Landsat 8 and Active Optical Sensor at Field Scale. Remote Sens. Appl. Soc. Environ..

[43] Kizilgeci F., Yildirim M., Islam M.S., Ratnasekera D., Iqbal M.A., Sabagh A.E. (2021). Normalized Difference Vegetation Index and Chlorophyll Content for Precision Nitrogen Management in Durum Wheat Cultivars under Semi-Arid Conditions. Sustainability.

[44] Casadesús J., Kaya Y., Bort J., Nachit M.M., Araus J.L., Amor S., Ferrazzano G., Maalouf F., Maccaferri M., Martos V. (2007). Using Vegetation Indices Derived from Conventional Digital Cameras as Selection Criteria for Wheat Breeding in Water-Limited Environments. Ann. Appl. Biol..

[45] Rehman T.H., Borja Reis A.F., Akbar N., Linquist B.A. (2019). Use of Normalized Difference Vegetation Index to Assess N Status and Predict Grain Yield in Rice. Agron. J..

[46] Vergara-Díaz O., Zaman-Allah M.A., Masuka B., Hornero A., Zarco-Tejada P., Prasanna B.M., Cairns J.E., Araus J.L. (2016). A Novel Remote Sensing Approach for Prediction of Maize Yield Under Different Conditions of Nitrogen Fertilization. Front. Plant Sci..

[47] Segarra J., Rezzouk F.Z., Aparicio N., González-Torralba J., Aranjuelo I., Gracia-Romero A., Araus J.L., Kefauver S.C. (2022). Multiscale Assessment of Ground, Aerial and Satellite Spectral Data for Monitoring Wheat Grain Nitrogen Content. Inf. Process. Agric..

[48] Kim W., Iizumi T., Nishimori M. (2019). Global Patterns of Crop Production Losses Associated with Droughts from 1983 to 2009. J. Appl. Meteor. Climatol..

[49] Yin H., Cao Y., Marelli B., Zeng X., Mason A.J., Cao C. (2021). Soil Sensors and Plant Wearables for Smart and Precision Agriculture. Adv. Mater..

[50] Coppedè N., Janni M., Bettelli M., Maida C.L., Gentile F., Villani M., Ruotolo R., Iannotta S., Marmiroli N., Marmiroli M. (2017). An in Vivo Biosensing, Biomimetic Electrochemical Transistor with Applications in Plant Science and Precision Farming. Sci. Rep..

[51] Reyns P., Missotten B., Ramon H., De Baerdemaeker J. (2002). A Review of Combine Sensors for Precision Farming. Precis. Agric..

[52] Shafi U., Mumtaz R., García-Nieto J., Hassan S.A., Zaidi S.A.R., Iqbal N. (2019). Precision Agriculture Techniques and Practices: From Considerations to Applications. Sensors.

[53] Armada-Moreira A., Dar A.M., Zhao Z., Cea C., Gelinas J., Berggren M., Costa A., Khodagholy D., Stavrinidou E. (2023). Plant Electrophysiology with Conformable Organic Electronics: Deciphering the Propagation of Venus Flytrap Action Potentials. Sci. Adv..

[54] Dufil G., Bernacka-Wojcik I., Armada-Moreira A., Stavrinidou E. (2022). Plant Bioelectronics and Biohybrids: The Growing Contribution of Organic Electronic and Carbon-Based Materials. Chem. Rev..

[55] Janni M., Claudia C., Federico B., Sara P., Filippo V., Nicola C., Manuele B., Davide C., Loreto F., Zappettini A. (2021). Real-Time Monitoring of Arundo Donax Response to Saline Stress through the Application of in Vivo Sensing Technology. Sci Rep.

[56] Vurro F., Janni M., Coppedè N., Gentile F., Manfredi R., Bettelli M., Zappettini A. (2019). Development of an In Vivo Sensor to Monitor the Effects of Vapour Pressure Deficit (VPD) Changes to Improve Water Productivity in Agriculture. Sensors.

[57] Vurro F., Manfredi R., Bettelli M., Bocci G., Cologni A.L., Cornali S., Reggiani R., Marchetti E., Coppedè N., Caselli S. (2023). In Vivo Sensing to Monitor Tomato Plants in Field Conditions and Optimize Crop Water Management. Precis. Agric.

[58] Vurro F., Marchetti E., Bettelli M., Manfrini L., Finco A., Sportolaro C., Coppedè N., Palermo N., Tommasini M.G., Zappettini A. (2023). Application of the OECT-Based In Vivo Biosensor Bioristor in Fruit Tree Monitoring to Improve Agricultural Sustainability. Chemosensors.

[59] Straitsresearch Tomato Market Size, Analysis, Report to 2031. https://straitsresearch.com/report/tomato-market.

[60] Sunera, Amna, Saqib S., Uddin S., Zaman W., Ullah F., Ayaz A., Asghar M., Rehman S., Munis M.F.H. (2020). Characterization and Phytostimulatory Activity of Bacteria Isolated from Tomato (Lycopersicon Esculentum Mill.) Rhizosphere. Microb. Pathog..

[61] Naeem M., Shahzad K., Saqib S., Shahzad A., Nasrullah, Younas M., Afridi M.I. (2022). The Solanum Melongena COP1LIKE Manipulates Fruit Ripening and Flowering Time in Tomato (Solanum Lycopersicum). Plant Growth Regul.

[62] Sivakumar R., Srividhya S. (2016). Impact of Drought on Flowering, Yield and Quality Parameters in Diverse Genotypes of Tomato (*Solanum Lycopersicum* L.). Adv. Hortic. Sci..

[63] Conti V., Romi M., Guarnieri M., Cantini C., Cai G. (2022). Italian Tomato Cultivars under Drought Stress Show Different Content of Bioactives in Pulp and Peel of Fruits. Foods.

[64] Alordzinu K.E., Li J., Lan Y., Appiah S.A., AL Aasmi A., Wang H., Liao J., Sam-Amoah L.K., Qiao S. (2021). Ground-Based Hyperspectral Remote Sensing for Estimating Water Stress in Tomato Growth in Sandy Loam and Silty Loam Soils. Sensors.

[65] Stutsel B., Johansen K., Malbéteau Y.M., Mccabe M.F. (2021). Detecting Plant Stress Using Thermal and Optical Imagery from an Unoccupied Aerial Vehicle. Front. Plant Sci..

[66] Zaman-Allah M., Vergara O., Araus J.L., Tarekegne A., Magorokosho C., Zarco-Tejada P.J., Hornero A., Albà A.H., Das B., Craufurd P. (2015). Unmanned Aerial Platform-Based Multi-Spectral Imaging for Field Phenotyping of Maize. Plant Methods.

[67] Gitelson A.A., Kaufman Y.J., Merzlyak M.N. (1996). Use of a Green Channel in Remote Sensing of Global Vegetation from EOS-MODIS. Remote Sens. Environ..

[68] Gitelson A., Merzlyak M.N. (1994). Quantitative Estimation of Chlorophyll-a Using Reflectance Spectra: Experiments with Autumn Chestnut and Maple Leaves. J. Photochem. Photobiol. B Biol..

[69] Rouse J.W., Haas R.H., Schell J.A., Deering D.W. (1973). Monitoring Vegetation Systems in the Great Plains with ERTS (Earth Resources Technology Satellite). NASA Spec. Publ..

[70] Idso S.B. (1982). Non-Water-Stressed Baselines: A Key to Measuring and Interpreting Plant Water Stress. Agric. Meteorol..

[71] Galieni A., D’Ascenzo N., Stagnari F., Pagnani G., Xie Q., Pisante M. (2021). Past and Future of Plant Stress Detection: An Overview from Remote Sensing to Positron Emission Tomography. Front. Plant Sci..

[72] Sun Z., Wang X., Wang Z., Yang L., Xie Y., Huang Y. (2021). Uavs as Remote Sensing Platforms in Plant Ecology: Review of Applications and Challenges. J. Plant Ecol..

[73] Almalki F.A., Soufiene B.O., Alsamhi S.H., Sakli H. (2021). A Low-Cost Platform for Environmental Smart Farming Monitoring System Based on iot and uavs. Sustainability.

[74] Wang T., Liu Y., Wang M., Fan Q., Tian H., Qiao X., Li Y. (2021). Applications of UAS in Crop Biomass Monitoring: A Review. Front Plant Sci.

[75] Fujiwara R., Kikawada T., Sato H., Akiyama Y. (2022). Comparison of Remote Sensing Methods for Plant Heights in Agricultural Fields Using Unmanned Aerial Vehicle-Based Structure from Motion. Front Plant Sci.

[76] Botta A., Cavallone P., Baglieri L., Colucci G., Tagliavini L., Quaglia G. (2022). A Review of Robots, Perception, and Tasks in Precision Agriculture. Appl. Mech..

[77] Zhang C., Valente J., Wang W., Guo L., Tubau Comas A., van Dalfsen P., Rijk B., Kooistra L. (2023). Feasibility Assessment of Tree-Level Flower Intensity Quantification from UAV RGB Imagery: A Triennial Study in an Apple Orchard. ISPRS J. Photogramm. Remote Sens..

[78] Cao X., Liu Y., Yu R., Han D., Su B. (2021). A Comparison of UAV RGB and Multispectral Imaging in Phenotyping for Stay Green of Wheat Population. Remote Sens..

[79] Shanmugapriya P., Latha K.R., Pazhanivelan S., Kumaraperumal R., Karthikeyan G., Sudarmanian N.S. (2022). Spatial Prediction of Leaf Chlorophyll Content in Cotton Crop Using Drone-Derived Spectral Indices. Curr. Sci..

[80] Mezera J., Lukas V., Horniaček I., Smutný V., Elbl J. (2022). Comparison of Proximal and Remote Sensing for the Diagnosis of Crop Status in Site-Specific Crop Management. Sensors.

[81] Baluja J., Diago M.P., Balda P., Zorer R., Meggio F., Morales F., Tardaguila J. (2012). Assessment of Vineyard Water Status Variability by Thermal and Multispectral Imagery Using an Unmanned Aerial Vehicle (UAV). Irrig Sci.

[82] Espinoza C.Z., Khot L.R., Sankaran S., Jacoby P.W. (2017). High Resolution Multispectral and Thermal Remote Sensing-Based Water Stress Assessment in Subsurface Irrigated Grapevines. Remote Sens..

[83] Khorsand A., Rezaverdinejad V., Asgarzadeh H., Majnooni-Heris A., Rahimi A., Besharat S., Sadraddini A.A. (2021). Linking Plant and Soil Indices for Water Stress Management in Black Gram. Sci. Rep..

[84] Ballester C., Brinkhoff J., Quayle W.C., Hornbuckle J. (2019). Monitoring the Effects of Water Stress in Cotton Using the Green Red Vegetation Index and Red Edge Ratio. Remote Sens..

[85] Katimbo A., Rudnick D.R., dejonge K.C., Lo T.H., Qiao X., Franz T.E., Nakabuye H.N., Duan J. (2022). Crop Water Stress Index Computation Approaches and Their Sensitivity to Soil Water Dynamics. Agric. Water Manag..

[86] Merilo E., Yarmolinsky D., Jalakas P., Parik H., Tulva I., Rasulov B., Kilk K., Kollist H. (2018). Stomatal VPD Response: There Is More to the Story Than ABA. Plant Physiol..

[87] Rossini M., Panigada C., Cilia C., Meroni M., Busetto L., Cogliati S., Amaducci S., Colombo R. (2015). Discriminating Irrigated and Rainfed Maize with Diurnal Fluorescence and Canopy Temperature Airborne Maps. ISPRS Int. J. Geo-Inf..

[88] Liu J., Li S., Yang X., Wei Z., Liu F. (2022). Effects of Soil Drought and Vapour Pressure Deficit (VPD) on Water Use Efficiency of Tomato Plants with Contrasting Endogenous ABA Levels. Sci. Hortic..

[89] Song X., Miao L., Jiao X., Ibrahim M., Li J. (2022). Regulating Vapor Pressure Deficit and Soil Moisture Improves Tomato and Cucumber Plant Growth and Water Productivity in the Greenhouse. Horticulturae.

[90] Ahi Y., Orta H., Gündüz A., Gültaş H.T. (2015). The Canopy Temperature Response to Vapor Pressure Deficit of Grapevine Cv. Semillon and Razaki. Agric. Agric. Sci. Procedia.

[91] Perez-Martin A., Flexas J., Ribas-Carbó M., Bota J., Tomás M., Infante J.M., Diaz-Espejo A. (2009). Interactive Effects of Soil Water Deficit and Air Vapour Pressure Deficit on Mesophyll Conductance to CO2 in Vitis Vinifera and Olea Europaea. J. Exp. Bot..

[92] Yuan W., Zheng Y., Piao S., Ciais P., Lombardozzi D., Wang Y., Ryu Y., Chen G., Dong W., Hu Z. (2019). Increased Atmospheric Vapor Pressure Deficit Reduces Global Vegetation Growth. Sci. Adv..

[93] Fu G., Shen Z.X. (2016). Environmental Humidity Regulates Effects of Experimental Warming on Vegetation Index and Biomass Production in an Alpine Meadow of the Northern Tibet. PLOS ONE.

[94] Kirnak H., Irik H.A., Unlukara A. (2019). Potential Use of Crop Water Stress Index (CWSI) in Irrigation Scheduling of Drip-Irrigated Seed Pumpkin Plants with Different Irrigation Levels. Sci. Hortic..

