# Supplementary material for: Field Plant Monitoring from Macro to Micro Scale: Feasibility and Validation of Combined Field Monitoring Approaches from Remote to in Vivo to Cope with Drought Stress in Tomato
Source: Plants (Basel). 2023 Nov 14;12(22):3851. doi: 10.3390/plants12223851 (PMC10674827; doi:10.3390/plants12223851)
Supplement: Supplementary file 1 [file plants-12-03851-s001.zip › plants-2628411-supplementary.pdf]

Supplementary Materials

Field Plant Monitoring from Macro to Micro Scale: Feasibility and Validation of Combined Field Monitoring Approaches from Remote to in Vivo to Cope with Drought Stress in Tomato

Filippo Vurro [1], Michele Croci [2], Giorgio Impollonia [2,*], Edoardo Marchetti [1], Adrian Gracia-Romero [3,4], Manuele Bettelli [1], José Luis Araus [3], Stefano Amaducci [2] and Michela Janni [1,*]

[1] Istituto dei Materiali per l'Elettronica e il Magnetismo (IMEM-CNR), Parco Area delle Scienze 37/A, 43124 Parma, Italy; filippo.vurro@imem.cnr.it (F.V.); manuele.bettelli@imem.cnr.it (M.B.)
[2] Department of Sustainable Crop Production, Università Cattolica del Sacro Cuore, Via Emilia Parmense, 84, 29122 Piacenza, Italy; michele.croci@unicatt.it (M.C.); stefano.amaducci@unicatt.it (S.A.)
[3] Integrative Crop Ecophysiology Group, Agrotecnio—Center for Research in Agrotechnology, Plant Physiology Section, Faculty of Biology, University of Barcelona, 08028 Barcelona, Spain; adrian.gracia@irta.cat (A.G.-R.); jaraus@ub.edu (J.L.A.)
[5] Field Crops Program, Institute for Food and Agricultural Research and Technology (IRTA), 251981 Lleida, Spain
* Correspondence: giorgio.impollonia@unicatt.it (G.I.); michela.janni@imem.cnr.it (M.J.)

Table S1. Field operations performed in 2019.

DAT	Operation
30	Bioristor insertion
36	AMNITRA 34.3 ($NH_4^+NO_3^-$)
43	AMNITRA 34.3 ($NH_4^+NO_3^-$)
43	Differential irrigation
53	KNO_3 13-0-46
53	Soil probe placement
56	UAV flight
62	UAV flight
63	KNO_3 13-0-46
63	Ridomil Gold R WG<br>Quantum R-OK
71	Treatment for noctuids
72	K_2SO_4
82	UAV flight
90	Harvest

Table S2. Vegetation and bioristor based indices. Green highlight the Micro ground/plant scale, Blight Blue the medium proximal scale, gray and yellow the macro aerial scale.

Scales	Index	Index Description	Bioristor Based Index	RGB Based Index	Thermal Based Index	Multispectral Based Index	References
Micro ground/plant	R	Sensor response					[13,50,58]
Medium Proximal	GA	Green Area					[28]
Medium Proximal	GGA	Greener Area					[28]
Medium Proximal	CSI	Crop Stress Index					[28]
Macro Aerial	CWSI	Crop Water Stress Index					[70,83]
Macro Aerial	GNDVI	Green Normalized Difference Vegetation Index					[67]
Macro Aerial	NDRE	Normalized Difference Red Edge Index					[68]
Macro Aerial	NDVI	Normalized Difference Vegetation Index					[69]

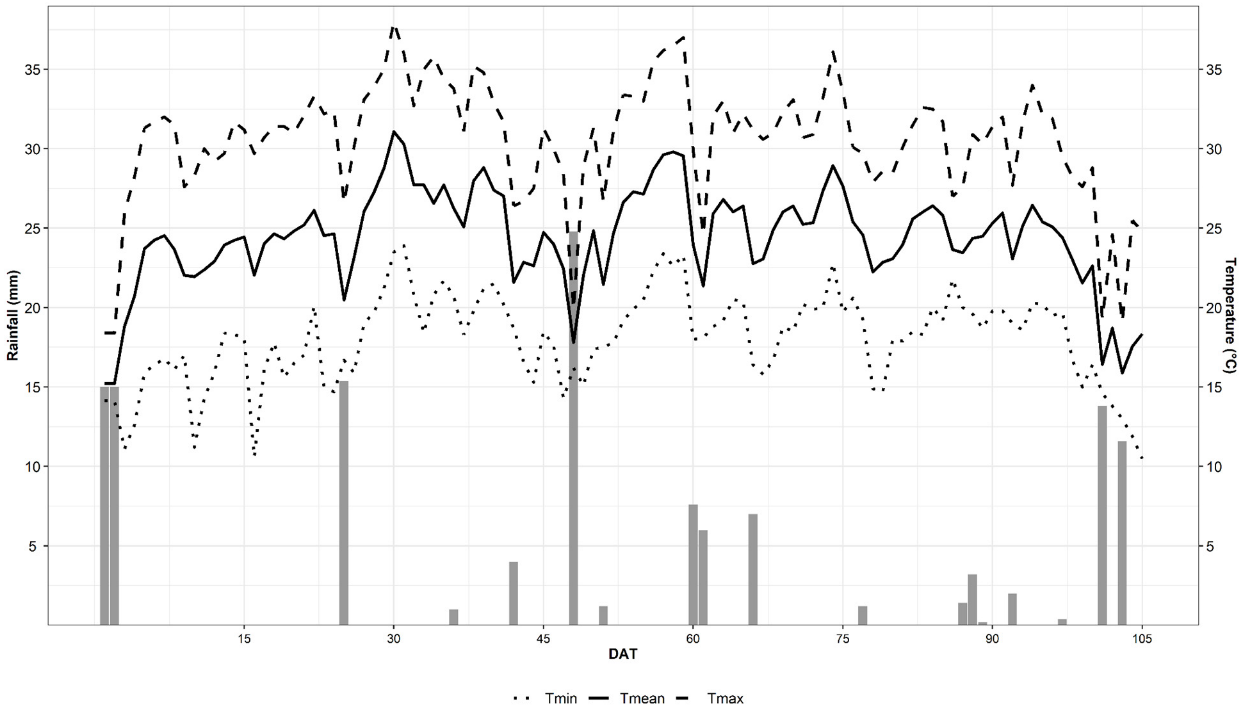

Figure S1. Environmental measurements. Daily rainfall, Mean Temperature, Temperature minimum (Tmin °C), Temperature maximum (Tmax °C).

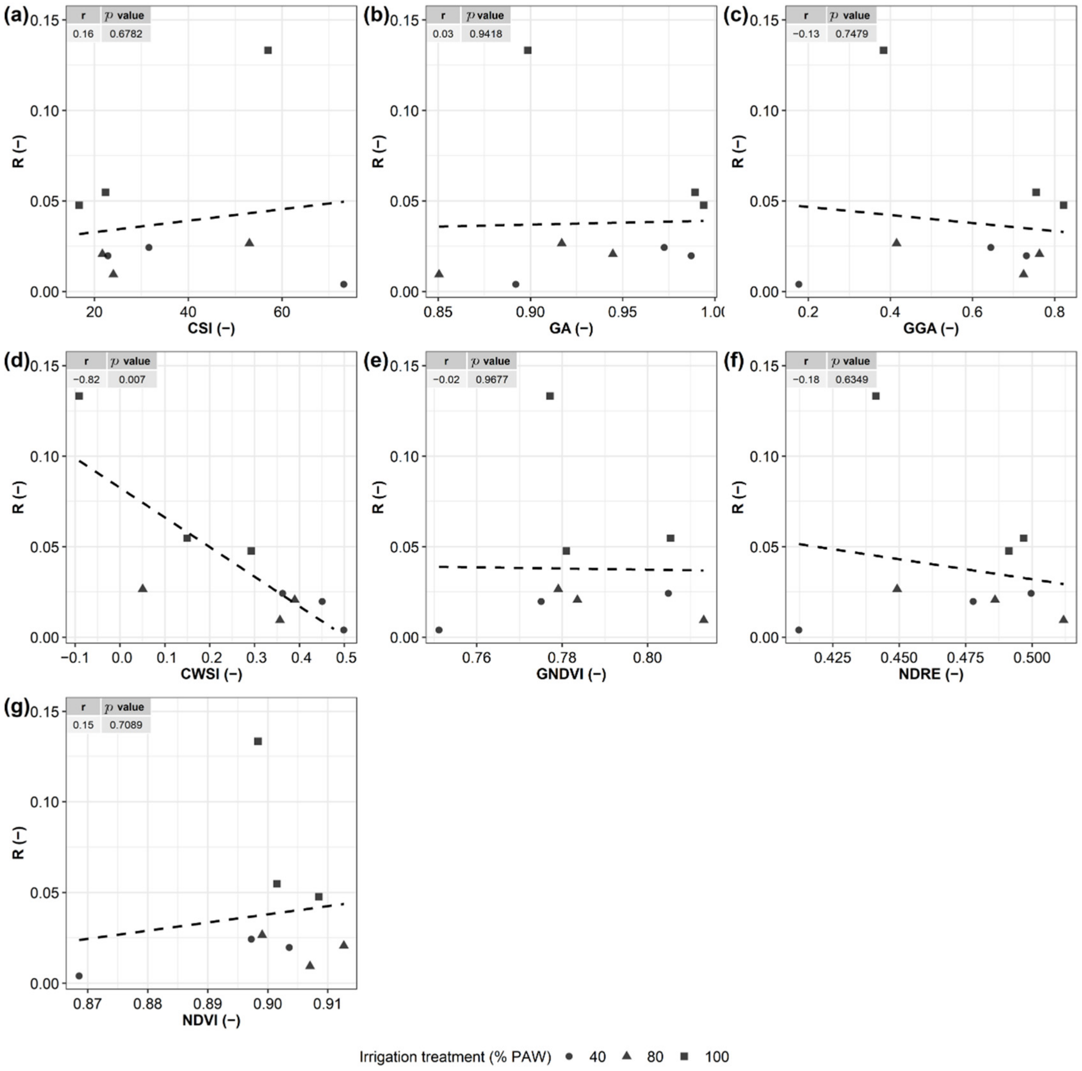

Figure S2. Correlation Plot between multiscale-acquired indices. Sensor response (R) and (**a**) CSI, (**b**) GA (**c**) GGA, (**d**) CWSI, (**e**) GNDVI, (**f**) NDRE, (**g**) NDVI.

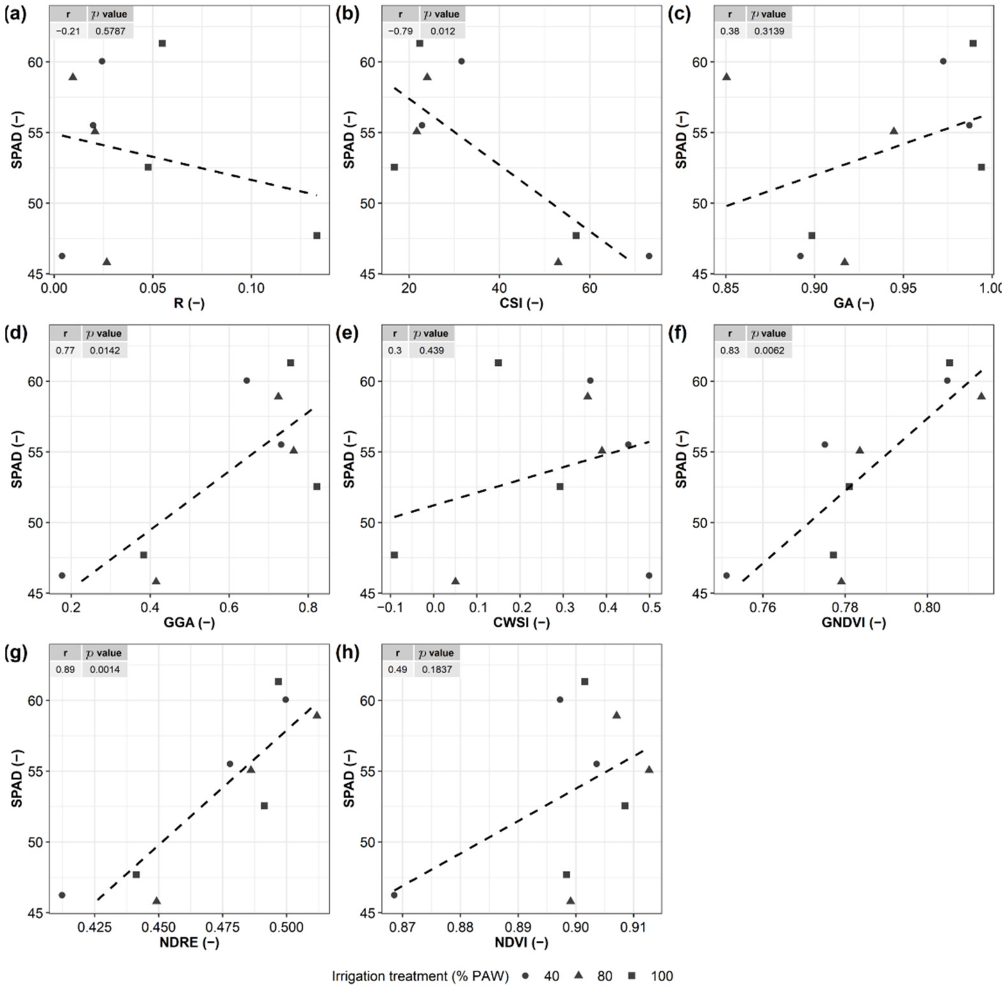

Figure S3. Correlation plot between the SPAD index and (**a**) R, (**b**) CSI, (**c**) GA (**d**) GGA, (**e**) CWSI, (**f**) GNDVI, (**g**) NDRE, (**h**) NDVI.

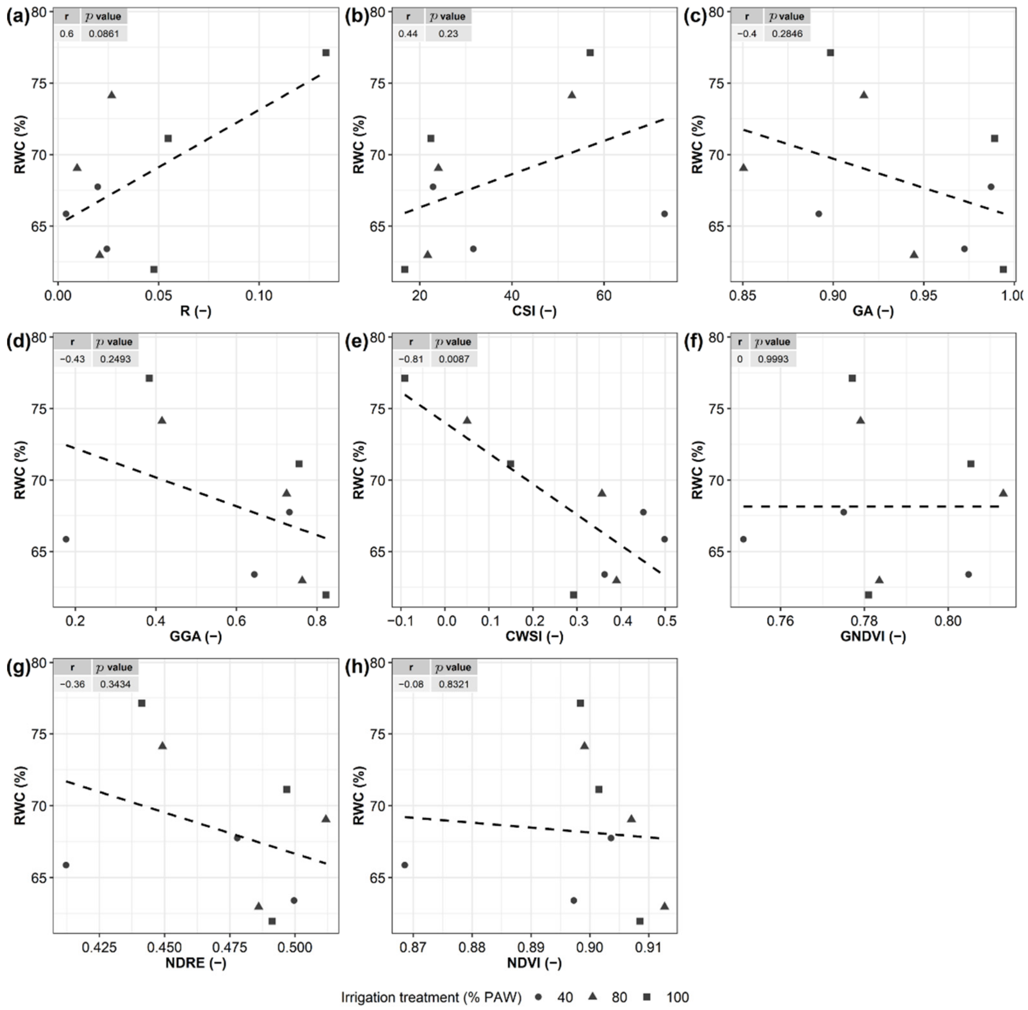

Figure S4. Correlation plot between the RWC and (**a**) R, (**b**) CSI, (**c**) GA (**d**) GGA, (**e**) CWSI, (**f**) GNDVI, (**g**) NDRE, (**h**) NDVI.
